# Qingrequzhuo capsule alleviated methionine and choline deficient diet-induced nonalcoholic steatohepatitis in mice through regulating gut microbiota, enhancing gut tight junction and inhibiting the activation of TLR4/NF-κB signaling pathway

**DOI:** 10.3389/fendo.2022.1106875

**Published:** 2023-01-19

**Authors:** Shuquan Lv, Zhongyong Zhang, Xiuhai Su, Wendong Li, Xiaoyun Wang, Baochao Pan, Hanzhou Li, Hui Zhang, Yuansong Wang

**Affiliations:** ^1^ Department of Endocrinology, Cangzhou Hospital of Integrated Traditional Chinese Medicine and Western Medicine of Hebei Province Affiliated to Hebei University of Chinese Medicine, Cangzhou, China; ^2^ Graduate School, Hebei University of Chinese Medicine, Shijiazhuang, China; ^3^ Graduate School, Chengde Medical University, Chengde, China

**Keywords:** nonalcoholic steatohepatitis, Qingrequzhuo capsule, methionine and choline deficient diet, gut microbiota, TLR4/NF-κB signaling pathway

## Abstract

Qingrequzhuo capsule (QRQZ), composed of Morus alba L., Coptis chinensis Franch., Anemarrhena asphodeloides Bunge, Alisma plantago-aquatica subsp. orientale (Sam.) Sam., Citrus × aurantium L., Carthamus tinctorius L., Rheum palmatum L., Smilax glabra Roxb., Dioscorea oppositifolia L., Cyathula officinalis K.C.Kuan, has been used to treat nonalcoholic steatohepatitis (NASH) in clinic. However, the mechanism of QRQZ on NASH remains unclear. Recent studies have found that the dysfunction of gut microbiota could impair the gut barrier and induce the activation of TLR4/NF-kB signaling pathway, and further contribute to the inflammatory response in NASH. Modulating the gut microbiota to reduce inflammation could prevent the progression of NASH. In this study, a mouse model of NASH was generated by methionine and choline deficient diet (MCD) and treated with QRQZ. First, we evaluated the therapeutic effects of QRQZ on liver injury and inflammation in the NASH mice. Second, the changes in the gut microbiota diversity and abundance in each group of mice were measured through 16S rRNA sequencing. Finally, the effects of QRQZ on gut mucosal permeability, endotoxemia, and liver TLR4/NF-kB signaling pathway levels were examined. Our results showed that QRQZ significantly reduced the lipid accumulation in liver and the liver injury in NASH mice. In addition, QRQZ treatment decreased the levels of inflammatory cytokines in liver. 16S rRNA sequencing showed that QRQZ affected the diversity of gut microbiota and a f f e c t e d t h e r e l a t i v e a b u n d a n c e s o f D u b o s i e l l a , Lachnospiraceae_NK4A136_group, and Blautiain NASH mice. Besides, QRQZ could increase the expression of tight junction proteins (zonula occludens-1 and occludin) in gut and decrease the lipopolysaccharide (LPS) level in serum. Western blot results also showed that QRQZ treatment decreased the protein expression ofTLR4, MyD88 and the phosphorylation of IkB and NF-kBp65 and qPCR results showed that QRQZ treatment down-regulated the gene expression of interleukin (IL)-1b, IL-6, and tumor necrosis factor (TNF)-a in liver. In conclusion, our study demonstrated that QRQZ could reduce the lipid accumulation and inflammatory response in NASH model mice. The mechanisms of QRQZ on NASH were associated with modulating gut microbiota, thereby inducing the tight junction of gut barrier, reducing the endotoxemia and inhibiting the activation of TLR4/NFkB signaling pathway in liver.

## Introduction

Non-alcoholic fatty liver disease (NAFLD) is one of the most common chronic liver diseases. The incidence of NAFLD has increased in recent years with the changing lifestyle and diet structure in China, thereby making NAFLD the second major liver disease after viral hepatitis. NAFLD may progress to liver fibrosis, cirrhosis, and even malignant transformation in the long term (1). During NAFLD development, nonalcoholic steatohepatitis (NASH) progresses to liver fibrosis and cirrhosis, and the interventions for NASH become a break point for NAFLD treatment. Currently, no specific treatment exists, and the disease is clinically managed through diet modification and increased exercise as the basic treatment of weight control and fat loss; however, a combined administration of drugs is needed to achieve comprehensive treatment (2). Therefore, the development of safe and effective drugs to delay disease progression has garnered considerable attention as a hot topic of research in this field.

Many studies have shown that traditional Chinese medicine (TCM) has a favorable therapeutic effect on NAFLD and NASH (3). A clinical randomized multicenter controlled study showed that Lingguizhugan decoction significantly improved dyslipidemia and insulin resistance in patients with NAFLD (4). In addition, a propensity score-matched cohort study confirmed that TCM intervention was effective in preventing the progression to cirrhosis in patients with NAFLD and NASH (5). A meta-analysis also confirmed the significant advantages of TCM in improving abnormal liver function and dyslipidemia in NASH patients (6). Elucidating the mechanism of action of TCM in the treatment of NAFLD and NASH can greatly facilitate the modernization of TCM and is becoming a hot spot in for researchers.

Gut microbiota are a group of microorganisms that colonize in the gut and have a symbiotic relationship with the host. They are large in number, with a total of 10–100 trillion in number, and dominated by bacteria while including archaea, fungi, protozoa, and viruses (7). The number and types of bacterial strains are in dynamic balance, which is a part of the body’s internal homeostasis. Bacterial abundance and compositional complexity increase from the stomach to the colon, showing increasing diversity, and are influenced by genetics, age, lifestyle, medications, and diet (8). These microbes have a symbiotic relationship with the human body, and play an important role in food digestion and nutrient absorption, such as decomposing complex polysaccharides, synthesizing multiple vitamins, and participating in the enterohepatic circulation of bile acids (9). A large number of studies have reported that patients with NAFLD have altered gut microbiota or dysbiosis, and significantly higher amounts of γ-proteobacteria and *Prevotella* have been identified in stool samples from children with NAFLD (10). In the NAFLD group, the *Firmicutes*/*Bacteroides* (*F/B*) ratio was lower than that in the healthy population, whereas the proportion of *Lactobacillus* spp. was higher in the healthy population, suggesting a disproportionate ratio between the species (11). An increase in fecal *Bacteroides*, *Escherichia coli*, and *Ruminococcus* and a decrease in beneficial bacteria such as *Lactobacillus* and *Bifidobacterium* is observed in patients with NASH (12). Regulation of gut microbiota has facilitated in opening new avenues of research for the treatment of NASH. Modulation of gut microbiota by butyrate alleviated high-fat diet (HFD)-induced NASH by improving gut mucosal permeability (13). Modulation of gut microbiota by indole-3-propionic acid promoted gut mucosal tight junctions, which consequently inhibited the liver inflammatory response in NASH mice (14) Soyasaponin A_2_ alleviated methionine and choline-deficient diet (MCD)-induced abnormalities in lipid metabolism in NASH mice by regulating gut microbiota and bile acid metabolism (15). Further investigation revealed that the imbalance of gut microbiota may lead to changes in the permeability of the gut mucosa, which subsequently leads to the entry of bacterial metabolites including lipopolysaccharide (LPS) from the intestine into the liver through the peripheral blood. This activates the toll-like receptor 4 (TLR4)/nuclear factor kappa B (NF-κB) signaling pathway and causes an inflammatory response in the liver, and inhibition of the inflammatory response by regulating the bacterial groups effectively improved NAFLD and NASH (16). Many studies have also demonstrated the regulatory effects of TCM on gut microbiota. Jianganxiaozhi formula improved the liver inflammatory response in NAFLD rats by regulating the abundance of *Alloprevotella*, *Lactobacillus*, and *Turicibacter* in the gastrointestinal tract to reduce the gut mucosal permeability in rats (17). Qiwei Baizhu Powder can treat diarrhea\ through promoting the proliferation of *Lactobacillus* and downregulating the abundances of *Proteus*, *Clostridium*, *Eubacterium*, *Facklamia*, and *Escherichia* (18–20). The combination of *Scutellaria baicalensis* Georgi and *Coptis chinensis* Franch could increase the abundance of short-chain fatty acids (SCFAs)-producing bacteria such as *Bacteroidales S24-7 group_norank*, *Parasutterella*, *Prevotellaceae UCG-001*, *Ruminiclostridium*, and *Ruminiclostridium* in type 2 diabetes mellitus (T2DM) rats (21).

Qingrequzhuo capsule (QRQZ), composed of *Morus alba* L., *Picrorhiza kurroa* Royle ex Benth., *Anemarrhena asphodeloides* Bunge, *Alisma plantago-aquatica subsp. orientale* (Sam.) Sam., *Citrus × aurantium* L., *Carthamus tinctorius* L., *Rheum officinale* Baill., *Smilax glabra* Roxb., *Dioscorea oppositifolia* L., and *Cyathula officinalis* K.C.Kuan, has shown in clinical studies to improve glucose metabolism measures, reduce the level of inflammatory cytokines and improve vascular endothelial function in patients with T2DM and NAFLD, with a good safety profile in treatment (22) However, the underlying mechanism of action remains unclear. In this study, a mouse model of NASH was generated by MCD and treated with QRQZ. First, we evaluated the therapeutic effects of QRQZ on liver injury and inflammatory response in the NASH mice. Second, the changes in the gut microbiota diversity and abundance in each group of mice were measured through 16S rRNA sequencing. Finally, the effects of QRQZ on gut mucosal permeability, endotoxemia, and liver TLR4/NF-κB signaling pathway levels were examined. This study was aimed to explore the effects of QRQZ on improving gut mucosal permeability by regulating gut microbiota, and its mechanism in the inhibition of the TLR4/NF-κB signaling pathway activation to alleviate NASH.

## Materials and methods

### Reagents

MCD was purchased from Sibeifu Bioscience Co., Ltd. (Beijing, China). Polyene phosphatidylcholine (PPC) was purchased from Sanofi. (Beijing, China). QRQZ was purchased from Cangzhou Hospital of Integrated Traditional Chinese Medicine and Western Medicine of Hebei Province. Triglyceride (TG, cat: A110-1-1), total cholesterol (TC, cat: A111-1-1), alanine aminotransferase (ALT, cat: C009-1-1), aspartate aminotransferase (AST, cat: C0101-2-1), superoxide dismutase (SOD, cat: A001-3-2), methane dicarboxylic aldehyde (MDA, cat: A003-1-2), and glutathione peroxidase (GSH-Px, cat: A006-2-1) biochemical test kits were obtained from Jiancheng Biological Engineering Institute (Nanjing, China). Enzyme-linked immunosorbent assay (ELISA) kits of mouse tumor necrosis factor alpha (TNF-α, cat: ml002095), interleukin (IL)-1β (cat: ml301814), IL-6 (cat: ml063159), LPS (cat: ml037221-2) were purchased from Enzyme-linked Biotechnology Co., Ltd. (Shanghai, China). Antibodies for zonula occludens-1 (ZO-1, cat:61-7300) and occludin (cat: 71-1500) were purchased from Thermo Fisher Scientific. Antibodies for TLR4 (cat: 19811-1-AP) and myeloid differentiation primary response 88 (MyD88, cat: 67969-1-Ig) were purchased from Proteintech Group, Inc. Antibodies for nuclear factor of kappa light polypeptide gene enhancer in B-cells inhibitor (IκB, cat: ab32518), phospho-IκB (p-IκB, cat: ab133462) were purchased from Abcam. Antibodies for nuclear factor kappa-light-chain-enhancer of activated B cells p65 subunit (NF-κBp65, cat: #8242), and phospho-NF-κBp65(p-NF-κBp65, cat: #3033) were purchased from Cell Signaling Technology.

### Animals

Sixty male C57BL/6 mice, weighing 20 ± 2g, were purchased from Beijing HFK Bioscience Co., Ltd. (SCXK 2021-0006). The feeding conditions of animals can be found in the **Supplementary Materials**. The animal study was approved by Ethics Committee of Hebei University of Chinese Medicine (Approval no. CZX2021-KY-026).

### Preparations of QRQZ

QRQZ was prepared by the pharmacy department of Cangzhou Hospital of Integrated Traditional Chinese and Western Medicine. Briefly, 15 g of *Morus alba* L., 9 g of *Coptis chinensis* Franch., 12 g of *Anemarrhena asphodeloides* Bunge, 12 g of *Alisma plantago-aquatica subsp. orientale* (Sam.) Sam., 15 g of *Citrus × aurantium* L., 9 g of *Carthamus tinctorius* L., 6g of *Rheum palmatum* L., 15 g of *Smilax glabra* Roxb., 12 g of *Dioscorea oppositifolia* L., 12 g of *Cyathula officinalis* K.C.Kuan were weighed, mixed, decocted and evaporated to obtain the extract powder of QRQZ. Then, the powders were made into capsule (0.5g per capsule) based on the medical institution preparation standard in Hebei (approval number: Z20050795).

### Animal grouping

After 1 week of acclimatization feeding, all mice were randomly divided into the control, model, PPC, QRQZ low-dose (LD-QRQZ), QRQZ middle-dose (MD-QRQZ), and QRQZ high-dose (HD-QRQZ) groups (n = 10 per group). The control group received normal diet and the remaining 5 experimental groups were administered the MCD (Sucrose 45.53%, Sodium Bicarbonate 0.75%, Corn Starch 15%, Maltodextrin 5%, Cellulose 3%, Corn Oil 10%, Multi Mineral S10001 3.5%, Multi Vitamin V10001 1%, Methionine 0%, Choline 0%, Alanine 0.35%, Arginine 1.21%, Asparagine 0.6%, Winterine 0.35%, Cystine 0.35%, glutamic acid 0.4%, glycine 2.33%, histidine 0.45%, isoleucine 0.82%, leucine 1.11%, lysine 1.8%, phenylalanine 0.75%, proline 0.35%, serine 0.35%, threonine 0.82%, tryptophan 0.18%, tyrosine 0.5%, valine 0.82%). The PPC group was administered 88 mg/kg of PPC through oral gavage. The contents of QRQZ were collected and dissolved in saline to prepare QRQZ mixture. The mixture was treated to mice through the gavage. The LD-QRQZ group, MD-QRQZ group and HD-QRQZ group received orally treatment of 0.48, 0.96, and 1.92 g/kg of QRZS respectively; and the control and model groups received an equal volume of saline as vehicle in parallel. All mice were gavaged once per day for 42 days. The mice were weighed and the weights were recorded every 7 days.

### Preparation of tissue samples

After 42 days of treatment, the mice were anesthetized with sodium pentobarbital. Blood was extracted from the heart, then the mice were sacrificed, and the serum was separated and stored at −80°C. The livers were quickly removed and weighed, and the liver tissues used for pathological sections in each group were isolated from the same location of the liver lobe and placed in 4% paraformaldehyde solution for fixation or quick-freezing storage. The remaining samples were quick-frozen and stored at −80°C. After careful extrusion of the cecum contents for collection, the colon was cut off and placed in 4% paraformaldehyde solution for fixation.

### Pathological staining

Paraffin sections were prepared from the 4% paraformaldehyde-fixed liver and colon tissues, and were observed under a light microscope after hematoxylin and eosin (HE) staining or sirius red staining. The NASH activity score (NAS) of liver was calculated as described previously (23, 24). The intestinal injury was evaluated by a 0–4 grading scale as previously reported (25). Besides, frozen liver tissues were sectioned and stained with oil red O. The positive area for oil red O and sirius red staining was quantified using Image Pro Plus 6.0 software based on the average optical density (AOD) (26).

### Biochemical testing

The liver was mixed with normal saline in the ratio of 1:9 (weight: volume), then ultrasonicated in an ice-water bath to completely lyse the homogenate, and centrifuged (2500 rpm, 10 min) to obtain the supernatant, which was 10% liver tissue homogenate. The concentrations of TG, TC, SOD, MDA, GSH-Px, and total protein were measured using commercial kits. Levels of HYP in liver tissue was tested to evaluate the fibrosis of liver. Serum concentrations of ALT and AST were measured using a commercial kit.

### ELISA

The concentrations of IL-1β, IL-6, and TNF-α in the liver tissue homogenate and the serum LPS level were measured using a double-antibody one-step sandwich ELISA. Briefly, the test sample, the standard and the detection antibody were added to the microplate wells coated with the capture antibody, and the plate was placed in a warm bath followed by thorough washing of the wells, after which the reaction substrate was added for color development. Absorbance was measured using a plate reader. The standard curve was plotted using the results of the standards, and the concentrations of the analytes in each sample were calculated.

### 16S rRNA sequencing

#### Fecal genomic DNA extraction

The total genomic DNA from the cecal contents of the mice was extracted with the cetyltrimethylammonium bromide (CTAB)/sodium dodecyl sulfate (SDS) method, and the DNA concentration and purity were measured with 1% agarose gel. DNA was diluted to 1 ng/µL with sterile water based on the concentration.

#### Polymerase chain reaction amplification and sequencing of 16S rRNA

The primers 338F (5′-ACTCCTACGGGAGGCAGCAG-3′) and 806R (5′-GGACTACHVGGGTWTCTAAT-3′) were used to amplify the V3 to V4 regions of the 16S rRNA gene. The PCR amplification system included 10 ng of template DNA, 0.2 µM of forward and reverse primers, and 15 µL Phusion^®^ High-Fidelity PCR Master Mix (New England Biolabs). The reaction conditions were as follows: pre-denaturation at 98°C for 1 minute, denaturation at 95°C for 10 seconds, annealing at 50°C for 30 seconds, and extension at 72°C for 30 seconds, for a total of 15 cycles; with a final holding at 72°C for 5 minutes. The mixture was then stored at 4°C. The mixed PCR products were purified with the Qiagen Gel Extraction Kit (Qiagen, Germany), and tested with 2% agarose gel electrophoresis. The sequencing library was generated using TruSeq^®^ DNA PCR-Free Sample Preparation Kit (Illumina, USA), and the library quality was assessed by Qubit@ 2.0 Fluorometer (Thermo Scientific) and Agilent Bioanalyzer 2100 system. Finally, the library was sequenced on the Illumina NovaSeq platform to obtain 250 bp of end-to-end sequences.

### Sequencing data analysis

The raw sequencing data were assembled and quality-controlled with FLASH (V1.2.7, http://ccb.jhu.edu/software/FLASH/) to obtain the final effective tags. The tags were clustered with Uparse (Uparse v7.0.1001, http://drive5.com/uparse/) at the 97% similarity level to obtain the operational taxonomic units (OTUs). The OTUs were annotated with taxonomic information against the Mothur algorithm-based Silva database (http://www.arb-silva.de/). The MUSCLE software (Version 3.8.31, http://www.drive5.com/muscle/) was used for multiple sequence alignment. The OTUs abundance information was normalized by the sequence number corresponding to the sample with the shortest sequence. Alpha diversity index and beta diversity analysis were subsequently performed. The Wilcoxon rank-sum test was used to test for inter-group differences in the diversity indices, the Kruskal–Wallis rank-sum test (Games–Howell was chosen as the *post-hoc* test) combined with the multiple testing method FDR were used to screen for differential bacteria, and a difference with *P* < 0.05 indicated statistical significance.

### Immunohistochemistry

Paraffin sections were made from the 4% paraformaldehyde-fixed colon tissues and placed in an incubator at 60°C overnight, with the endogenous peroxidase treated with methanol-hydrogen peroxide, followed by clearing with PBS and distilled water. After antigen recovery and blocking, the sections were stained immunohistochemically, with rabbit anti-ZO-1 (1:50) or rabbit anti-occludin (1:125) added dropwise to the sections, and incubated at 4°C overnight. After washing, the secondary antibody (1:10000) was added dropwise. The sections were then washed, mounted after color development and hematoxylin re-staining, and observed under a light microscope for expression of ZO-1 and occludin in the colon tissues. The expression in the positive regions was quantitatively analyzed using the Image Pro Plus 6.0 software based on the AOD (27).

### Reverse transcriptase quantitative PCR

The mRNA of *IL-1β, IL-6*, and *TNF-α* was determined for their hepatic expression with reverse transcriptase quantitative PCR (qPCR) after extraction of the total RNA from the liver tissues. The primer sequences were included in **Supplementary Materials**. The mRNA expression was relative to that of *β-actin*. The relative expression was calculated with the 2^−ΔΔCT^ method.

### Western blot

20 mg of liver tissues were weighed and added with 150 μL of RIPA lysis buffer, the homogenate was centrifuged, and the proteins were retained. The total protein concentration was determined with the bicinchoninic acid (BCA) assay. An equal amount of 10 μg of protein was collected from each sample for separation with sodium dodecyl sulfate–polyacrylamide gel electrophoresis (SDS-PAGE) under the following conditions: 90 V for 20 minutes and 130 V for 1 hour. The separated proteins after electrophoresis were then transferred to the PVDF membrane at 130 V and 300 mA for 2 hours, blocked with 5% skim milk powder at room temperature for 2 hours, and added with the rabbit anti-mouse primary antibodies of TLR4, MyD88, IκB, p-IκB, NF-κBp65, p-NF-κBp65 and β-actin at dilutions of 1:1000, 1: 1000, 1:1000, 1:1000, 1:1000, 1:1000, 1:1000, and 1:2000, respectively. The membranes were incubated overnight at 4°C. After the membranes were washed, the secondary antibody (goat anti-rabbit IgG diluted at 1:9000) was added, followed by incubation at room temperature for 2 hours. After the membranes were washed with tris-buffered saline with 0.1% tween 20 (TBST), enhanced chemiluminescence (ECL) reagents were added for development and detection, and the grayscale values of the bands were quantitatively analyzed with Image Pro Plus 6.0.

### Statistical analysis

Statistical analysis was performed with the statistical software SPSS 20.0, and data are expressed as mean ± standard deviation (SD). One-way analysis of variance (ANOVA) with Tukey’s HSD (honest significant difference) *post-hoc* test was used for intergroup comparisons. Spearman’s correlation analysis was used to evaluate the correlation of therapeutic indicators and changed gut microbiota. A difference with *P* < 0.05 indicated statistical significance.

## Results

### QRQZ treatment improved the liver injury and inflammation in NASH mice

**Figure 1:**
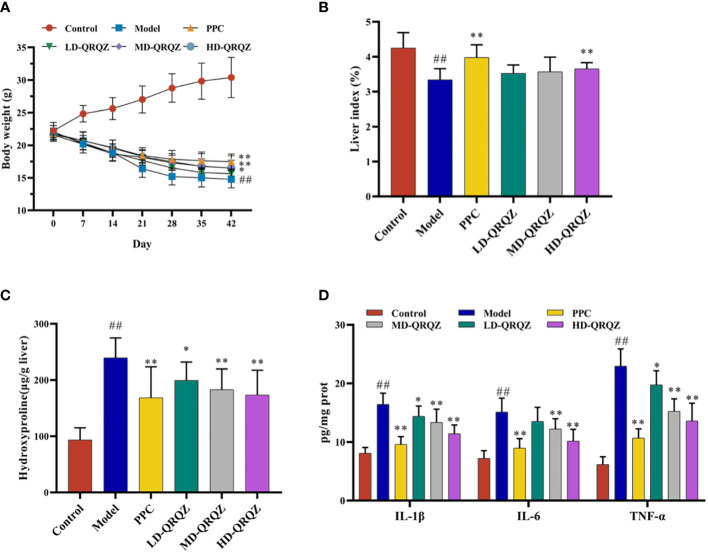
QRQZ alleviated liver injury and inflammation in MCD induced NASH mice. **(A)** Body weight change curves of each group. **(B)** The liver/body weight indexes of each group. **(C)** Hydroxyproline levels in liver of each group. **(D)** Serum pro-inflammatory cytokines of each group. Data are presented as the mean ± standard deviation. ##*P* < 0.01 as compared to the Control group; *P < 0.05 as compared to the Model group; **P < 0.01 as compared to the Model group.

**Figure 2:**
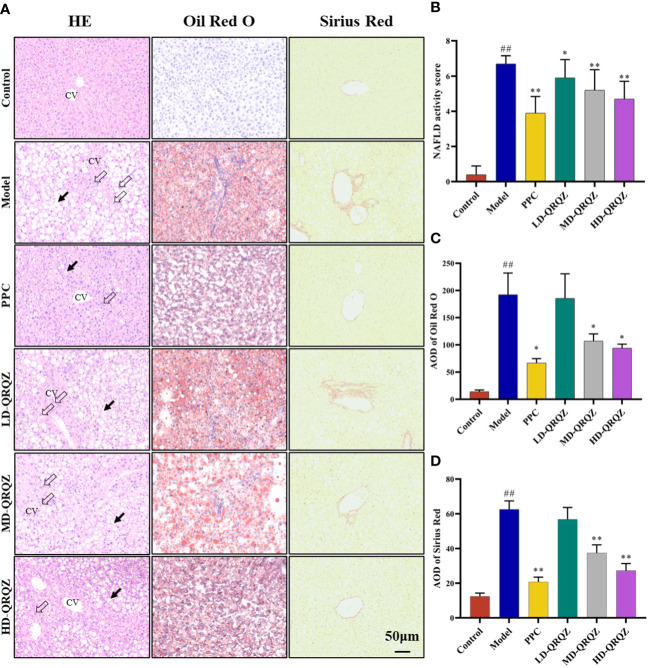
QRQZ alleviated hepatic pathological changes in MCD induced NASH mice. **(A)** HE staining, Oil Red O and Sirius Red staining of liver tissue. Black arrow indicated lipid accumulation. Hollow arrow indicated inflammatory foci. CV: Central vein. (200×). **(B-D)** NAS of HE staining of liver **(B)**. AOD of Oil Red O **(C)** and Sirius Red staining **(D)**. Data are presented as the mean ± standard deviation. ##*P* < 0.01 as compared to the Control group; *P < 0.05 as compared to the Model group; **P < 0.01 as compared to the Model group.

Mice treated with MCD for 42 days showed a significant decrease in body weight and liver index compared to the control group, whereas treatment with both PPC and HD-QRQZ increased body weight and liver index (**Figures 1A, B**). HYP test result showed that the HYP levels were higher in model group compared with the control. PPC and HD-QRQZ treatment lowered the concentration of HYP in liver (**Figure 1C**). HE staining showed that the model group had disorganized hepatic plate arrangement, inflammatory cell infiltration, and a large amount of vacuolar-like degeneration; Oil red O staining revealed that the liver in the model group had extremely severe steatosis, with huge fat droplets occupying most of the area; Sirius red staining showed increased collagen deposition in liver as expected. (**Figure 2A**); and PPC and QRQZ interventions alleviated the above pathological changes in different degrees. (**Figures 2B–D**).

In addition, several biochemical parameters were measured for a more comprehensive assessment on the liver injury. In terms of fat metabolism, TC and TG appeared to be significantly increased in the liver tissues of the model group compared to those in the control group; in terms of liver function, the serum ALT and AST activities were also significantly increased in the model group compared to those in the control group; and in terms of oxidative stress indicators, the SOD and GSH-Px activities decreased and the MDA levels increased in the model group compared to those in the control group. In contrast, these indicators were significantly improved in the PPC and HD-QRQZ groups (**Table 1**). The ELISA results showed that the inflammatory cytokines IL-1β, IL-6, and TNF-α were significantly increased in the liver tissues of the model group compared to those in the control group, and PPC significantly reduced the levels of these inflammatory cytokines. Besides, at different dosages, QRQZ reduced the levels of these inflammatory cytokines in a dose-dependent manner to varying degrees, and the effect was most significant in the HD-QRQZ group (**Figure 1D**).

**Table 1:** Changes in physiological indices, oxidative stress factors after QRQZ treatment.

	Biochemical Parameters	Control	Model	PPC	LD-QRQZ	MD-QRQZ	HD-QRQZ
**Liver lipid profile**	**TC** (μmol/g prot)	82.8 ± 21.8	161.6 ± 41.6^##^	93.9 ± 36.9^**^	113.7 ± 28.4^*^	92.6 ± 32.8^**^	91.9 ± 38.8^**^
	**TG** (μmol/g prot)	120.2 ± 27.4	817.0 ± 51.7^##^	309.8 ± 40.4^**^	721.2 ± 38.6^**^	581.0 ± 50.4^**^	514.3 ± 53.3^**^
**Liver function**	**ALT** (U/L)	31.4 ± 14.1	207.0 ± 27.8^##^	71.4 ± 33.3^**^	172.6 ± 45.0	143.6 ± 25.1^**^	99.7 ± 46.4^**^
	**AST** (U/L)	79.8 ± 24.6	216.7 ± 39.6^##^	134.2 ± 57.0^**^	178.1 ± 50.9	164.2 ± 56.3^*^	150.0 ± 42.9^**^
**Oxidative stress**	**SOD** (U/mg prot)	196.4 ± 19.4	101.5 ± 33.0^##^	166.0 ± 20.8^**^	129.5 ± 21.0^*^	146.2 ± 19.6^**^	153.6 ± 17.7^**^
	**MDA** (nmol/mg prot)	5.5 ± 1.0	21.0 ± 1.9^##^	10.8 ± 2.2^**^	16.1 ± 2.6^**^	13.7 ± 2.8^**^	12.6 ± 1.5^**^
	**GSH-Px** (U/mg prot)	48.2 ± 4.5	25.4 ± 3.1^##^	37.5 ± 1.7^**^	28.5 ± 2.0^*^	30.0 ± 2.9^**^	32.0 ± 3.3^**^

Control, Model, PPC, LD-QRQZ, MD-QRQZ and HD-QRQZ (n = 10 per group) groups. Data are presented as the mean ± standard deviation. ^##^P < 0.01 as compared to the Control group; ^*^P < 0.05 as compared to the Model group; ^**^P < 0.01 as compared to the Model group.

The above results showed that NASH was well induced through the administration of MCD to mice for 6 weeks, and that QRQZ had a therapeutic effect on NASH, which was most significant at the high dose. Therefore, the HD-QRQZ group was selected for the subsequent gut microbiota study.

### QRQZ treatment affected the gut microbiota in NASH mice

**Figure 3:**
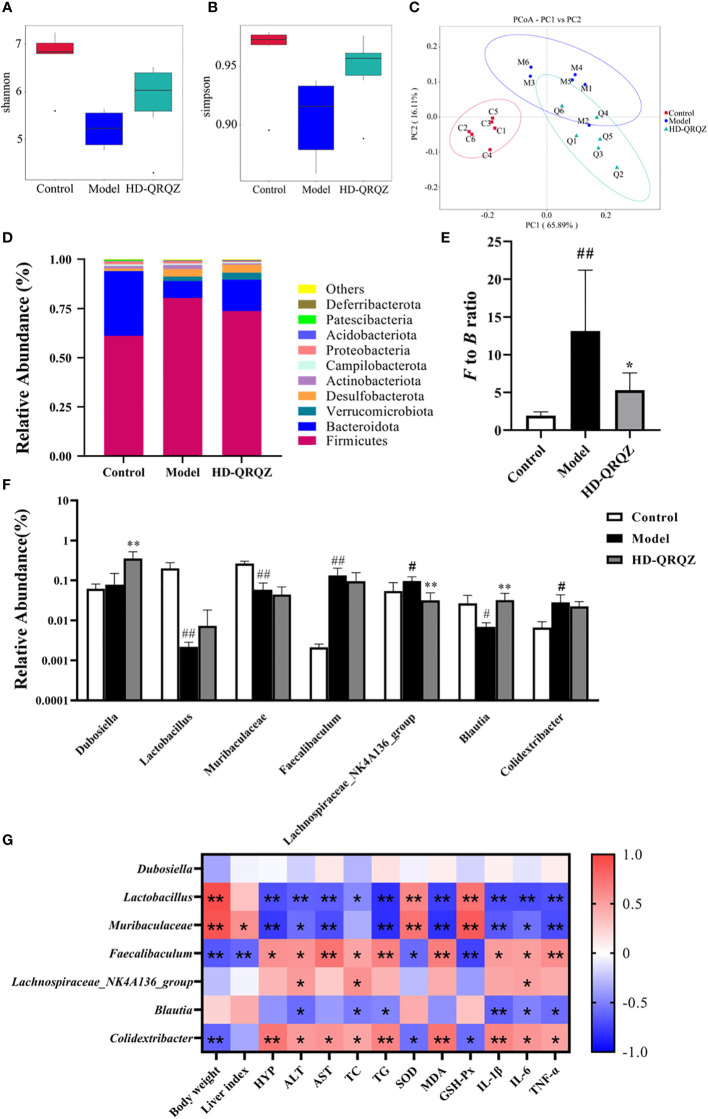
QRQZ treatment affected the gut microbiota community in Model mice. **(A, B)** Shannon and Simpson index was higher in HD-QRQZ group than that in the Model group. **(C)** PCoA indicated more similar beta diversity between HD-QRQZ and Control groups than that between the Model and Control groups (C: Control group; M: Model group; Q: HD-QRQZ group). **(D, E)** At the phylum level, QRQZ treatment decreased the *F* to *B* ratio in Model mice. **(F)** At the genus level, QRQZ treatment affected the relative abundances of *Dubosiella, Lachnospiraceae_NK4A136_group, Blautia*, in Model mice. **(G)** Correlation analysis of therapeutic indicators and changed gut microbiota using spearman’s analysis (heatmap). Color coding scale indicates the pearson correlation coefficient from heatmap, the deeper red or blue indicates the higher absolute of the value. *P < 0.05, **P < 0.01. Control, Model and HD-QRQZ (n = 6 per group) groups. Data are presented as the mean ± standard deviation. ^#^p < 0.05 as compared to the control group; ^##^ *P* < 0.01 as compared to the Control group; *P < 0.05 as compared to the Model group; **P < 0.01.

The 16S rRNA sequencing results were used to construct a clustering table for subsequent analysis of different groups of mouse gut microbiota. The diversity of the gut microbial community was assessed by calculating the Shannon and Simpson indices. The results showed that the Shannon index and Simpson index were decreased in the model group compared to those in the control group, indicating that NASH reduced the diversity of gut microbiota in the mice (**Figures 3A, B**). Then, we calculated the magnitude of differences in the microbial communities between different groups with the principal coordinate analysis (PCoA). The PCoA results showed that the sample points of the model group were significantly separated and distant from those of the control group, whereas the sample points of the HD-QRQZ group were well separated from those of the model group (**Figure 3C**).

As shown in **Figure 3D**, *Firmicutes* and *Bacteroidetes* were the dominant taxa in gut microbiota at the phylum level for each group. The *F/B* ratio was significantly increased in the model group compared to that in the control group, whereas the *F/B* ratio was significantly decreased after HD-QRQZ intervention (**Figure 3E**). At the genus level, the relative abundance of *Faecalibaculum*, *Lachnospiraceae_NK4A136_group*, and *Colidextribacter* was significantly increased in the model group compared to that in the control group, the relative abundance of *Lactobacillus*, *Muribaculaceae*, *Blautia* was significantly decreased; compared to the model group, the relative abundance of *Dubosiella* and *Blautia* was significantly increased in the HD-QRQZ group, and the relative abundance of *Lachnospiraceae_NK4A136_group* was significantly decreased (**Figure 3F**).

The correlation analysis showed that *Faecalibaculum* and *Colidextribacter* were positively correlated with the majority of NASH pathological indicators, oxidative stress indicators, and inflammatory cytokines while *Lactobacillus* and *Muribaculaceae* showed negatively correlation with most of the indicators (**Figure 3G**).

### QRQZ treatment reduced colonic permeability and alleviated endotoxemia in NASH mice

**Figure 4:**
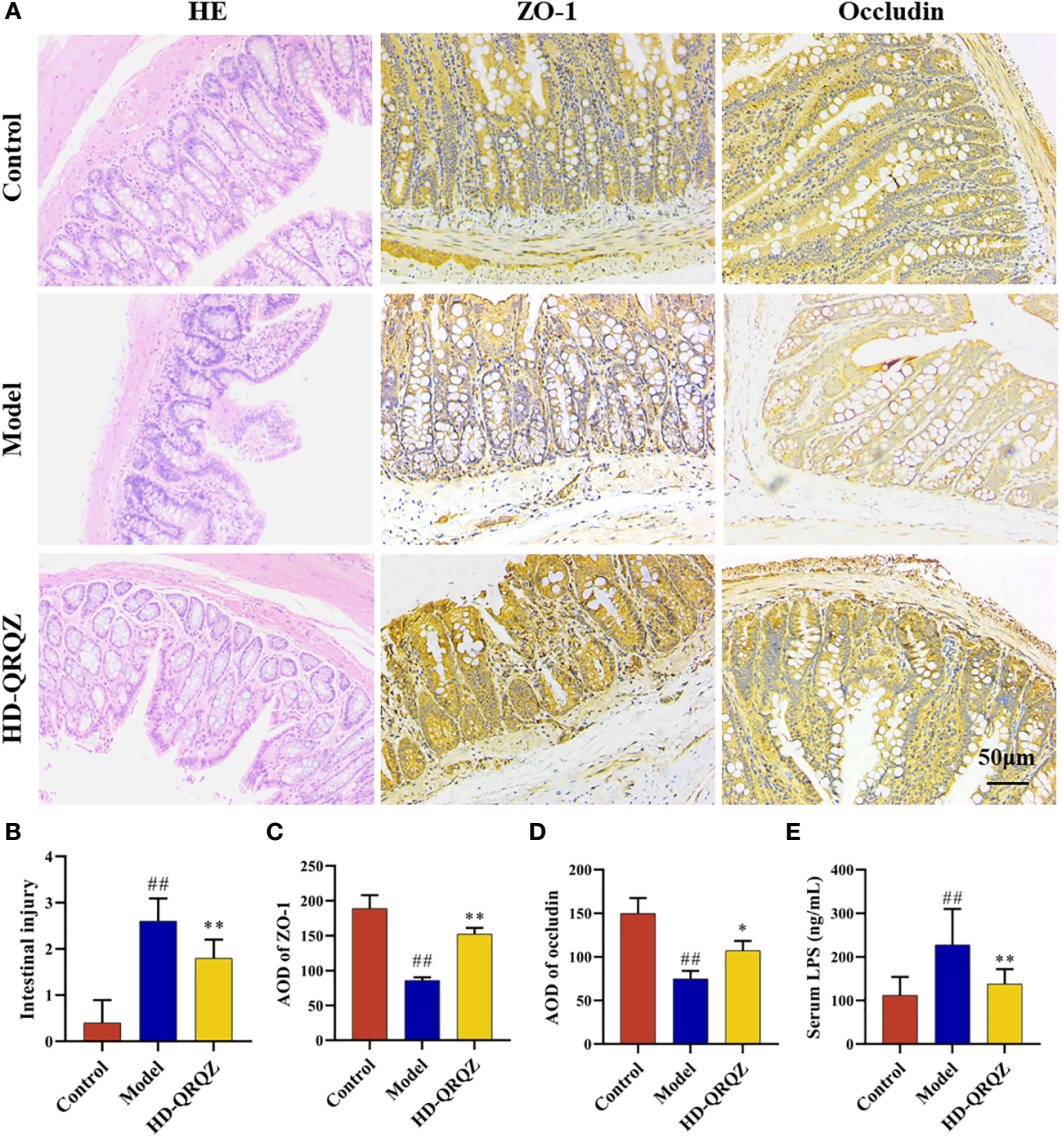
QRQZ improved the gut barrier. **(A)** HE staining of colon from each group and immunohistochemistry showed expression of ZO-1 and Occludin in colon. (200×) **(B)** Histological assessment of intestinal injury. **(C, D)** AOD of ZO-1 and Occludin. **(E)** Serum LPS levels of each group. Control, Model and HD-QRQZ (n = 6 per group) groups. Data are presented as the mean ± standard deviation. ^##^ *P* < 0.01 as compared to the Control group; *P < 0.05 as compared to the Model group; **P < 0.01 as compared to the Model group.

HE staining result showed incomplete arrangement of epithelial cells in colon can be observed in model group while HD-QRQZ alleviated the pathological changes in colon (**Figure 4A**). Likewise, the intestinal injury score was higher in model group compared with the control and HD-QRQZ treatment reduced the intestinal injury score in colon (**Figure 4B**). The effect of QRQZ on the intestinal expression levels of the tight junction proteins ZO-1 and occludin in the NASH mice was examined through immunohistochemical assays. The results showed that the areas of positive expression of ZO-1 and occludin were significantly reduced in the colonic tissues of mice in the model group compared to that in the control group. HD-QRQZ treatment elevated the positive areas of ZO-1 and occludin to different degrees in the mouse colonic tissues (**Figures 4A, C, D**). In addition, we assessed endotoxemia in each group of mice by measuring the LPS level in serum with ELISA, and the serum LPS level was significantly increased in the NASH mice compared to those in the control group, and it was reduced significantly after HD-QRQZ intervention (**Figure 4E**).

### QRQZ treatment inhibited the activation of TLR4/NF-κB signaling pathway in the liver of NASH mice

**Figure 5:**
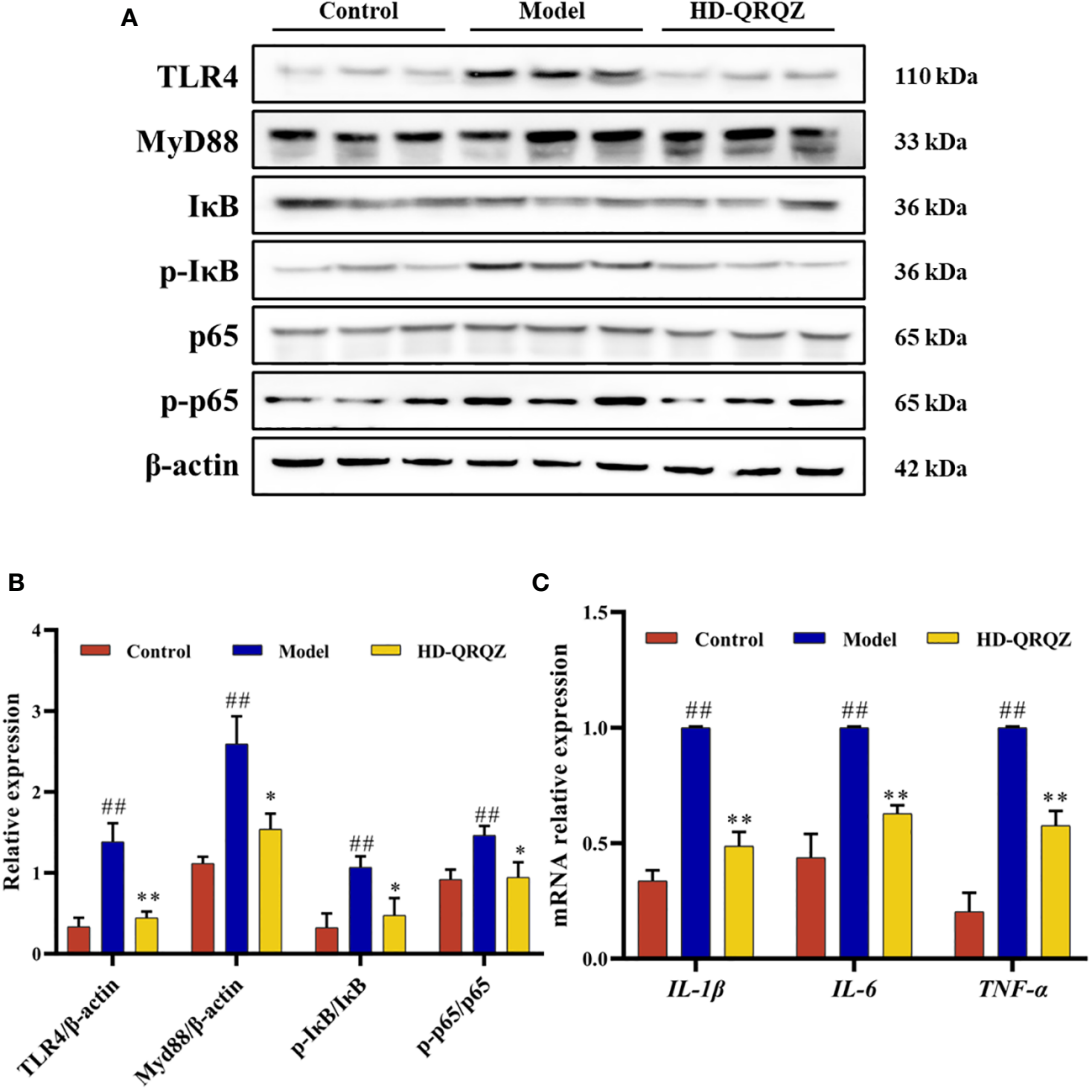
Effects of QRQZ on TLR4/NF-κB signaling pathway and mRNA expression of pro-inflammatory cytokines in liver. **(A, B)** The protein expression of TLR4, MyD88, IκB, p- IκB, p65, p- p65 in liver was detected by western blot. **(C)** mRNA expression of pro-inflammatory cytokines (*IL-1β*, *IL-6* and *TNF-α*) in liver was investigated using qPCR. Control, Model and HD-QRQZ (n = 3 per group) groups. Data are presented as the mean ± standard deviation. ^##^ *P* < 0.01 as compared to the Control group; *P < 0.05 as compared to the Model group; **P < 0.01 as compared to the Model group.

The levels of TLR4, MyD88, p-IκB, and p-NF-κBp65, which are important factors of the TLR4/NF-κB signaling pathway, were measured in liver with western blotting. The results showed that the protein levels of TLR4 and MyD88 and the phosphorylation levels of IκB and NF-κBp65 were significantly increased in the liver of the model group compared to those in the control group; however, after high-dose of QRQZ treatment, the TLR4 and MyD88 protein levels and IκB and NF-κBp65 phosphorylation levels were significantly reduced in the mouse liver (**Figures 5A, B**). In addition, qPCR was used to measure the mRNA expression of *IL-1β, IL-6*, and *TNF-α* in the liver, important downstream target genes of TLR4/NF-κB, and the results showed that the mRNA expression of *IL-1β, IL-6*, and *TNF-α* was upregulated in the liver tissues of the NASH mice compared to that in the control group. In contrast, high-dose of QRQZ treatment significantly downregulated the mRNA level of *IL-1β, IL-6*, and *TNF-α* in the liver of the NASH mice (**Figure 5C**).

## Discussion

In the present study, we generated NASH model mice based on MCD. The MCD is widely used in animal studies of NASH, and MCD-based models have replicated the histological features of steatohepatitis and fibrosis observed in human NASH, including steatosis, intralobular inflammation and hepatocyte ballooning (28). The advantage of choosing MCD for modeling is that it effectively and reproducibly induces liver steatosis and inflammation similar to human NASH histology in a relatively short period of time. Furthermore, male C57BL/6 mice show the most inflammation and necrosis as well as histological features that most similar to NASH (29). Although the MCD model lacks the key metabolic features of NASH, such as insulin resistance and obesity, which would lead to a significant decrease in the body mass of mice (28), it is still recognized as one of the major models of NASH because it manifests similar and equally severe histological features as human NASH (30). The generation mechanism of this model is deficiency of methionine and choline, which are necessary for hepatic β-oxidation and very-low-density lipoprotein (VLDL) production, whereas the lack of choline affects hepatic VLDL secretion, leading to lipid deposition in the liver. In addition, oxidative stress and changes in cytokines and adipocytokines can lead to liver damage (29). Our results also demonstrated a significant increase in the serum transaminase levels, as well as an increase in the liver TG level. Histological staining showed that the model mice had significant steatosis, fibrosis, and a large number of inflammatory cell infiltrates in the liver, which is consistent with the pathological changes of NASH. Liver fibrosis is also an important pathological feature of NASH, we also used Sirius red staining and tested the levels of HYP in liver to evaluate the liver fibrosis in each group. Our results showed that the collagen deposition and the levels of HYP were significantly increased in the NASH model mice. QRQZ significantly improved abnormal liver function in NASH mice while reducing hepatic lipid deposition and improving the liver fibrosis. Furthermore, studies have shown that *Morus alba* L. could inhibit lipogenesis in liver in obese mice (31). Hesperetin and Naringenin in *Citrus × aurantium* L could reduce hepatic steatosis NAFLD and NASH models (32, 33). *Rheum palmatum* L. could also ameliorate HFD-induced hepatic steatosis (34). These herbs and components may exert the major regulatory effects on hepatic steatosis. In addition, PPC was chosen as a positive control drug in this study, and PPC is commonly used in clinical practice for the treatment of NASH (35). The outcomes of the present study revealed that there was no significant difference in the improvement of liver function or liver lipid deposition between the MD-QRQZ and HD-QRQZ interventions and PPC, thereby suggesting that QRQZ can be used as an alternative to PPC in the treatment of NASH.

Oxidative stress is an important pathological process in NASH. The results of this study showed that QRQZ increased the SOD and GSH-Px activities and decreased the MDA level in the NASH liver tissues. Cytokines are important inflammatory markers in NASH, and studies have found elevated concentrations of IL-1β, IL-6, and TNF-α in NASH patients, with TNF-α being the most significant (36, 37). This study showed that IL-1β, IL-6, and TNF-α levels were significantly increased in the liver of the model group, and the elevation of TNF-α was the most significant, which are consistent with previous studies. All inflammatory cytokines decreased in varying degrees after QRQZ treatment, with the most significant decreases achieved after administering medium and high doses. Besides, coptisine from *Coptis chinensis* Franchcan has been demonstrated with anti-inflammatory effect through inhibiting the activation of NF-κB (38). *Citrus × aurantium* L. and its flavonoids possessed multiple bioactive potentials including anti-oxidative and anti-inflammatory activities (39, 40). Carthamus red from *Carthamus tinctorius* L. exerted anti-oxidative effect in CCl_4_-induced liver injury model rats (41). Previous studies have also demonstrated that *Smilax glabra* Roxb. and its active ingredients showed a wide range of pharmacological effects, including anti-inflammatory and anti-oxidative potentials (42). These herbs and components may exert the major anti-inflammatory and anti-oxidative effects in QRQZ.

Gut microbiota play an important role in NASH pathogenesis. In recent years, several pathways involving the gut–liver axis, gut barrier function, liver steatosis, and liver inflammation were suggested as the main mechanisms linking gut microbiota to NASH (43). Studies on the regulation of gut microbiota for treatment of NASH by antibiotic therapy and fecal transplantation have been performed in animals with favorable outcomes (44, 45), and clinical trials based on fecal transplantation are being conducted. The combined α- diversity and β-diversity in the 16S rRNA analysis in this study showed that the gut microbiota of the NASH model mice was severely disturbed, and the microbiota diversity and interpopulation differences were significantly changed, whereas the HD-QRQZ group showed higher population diversity, but failed to achieve a similar differential population structure to the control group. Changes in the *F/B* ratio are closely associated with many diseases. Although many studies have concluded that the *F/B* ratio is associated with obesity and NAFLD (46), fewer studies have associated the *F/B* ratio with NASH. This study showed that the *F/B* ratio in the NASH model group was significantly increased than that in the control group, whereas the ratio showed some improvement after treatment with HD-QRQZ, suggesting the *F/B* ratio as a potential reference for the diagnosis of NASH gut microbiota.

We also selected the top few bacteria with the greatest relative total abundance at the genus level for comparison. The relative abundance of *Faecalibaculum*, *Lachnospiraceae_NK4A136_group*, and *Colidextribacter* was significantly increased while the relative abundance of *Lactobacillus*, *Muribaculaceae*, *Blautia* was significantly decreased in the model group compared to that in the control group. HD-QRQZ could decrease the relative abundance of *Lachnospiraceae_NK4A136_group* and increase the relative abundance of *Dubosiella* and *Blautia*. *Dubosiella* abundance appeared to be negatively correlated with measures related to obesity and colonic inflammation (47–49). Several species under the genus *Lactobacillus* have been shown to improve lipid metabolism and inhibit lipid peroxidation (50, 51), and a clinical trial demonstrated that a mixed supplementation of several bacteria of the genus *Lactobacillus* improved liver fat accumulation and regulated gut microbiota (52). In this study, *Lactobacillus* has shown negative correlations with nearly all pathological indicators, suggesting that it may be a potentially beneficial bacterium. However, the results of this study found that QRQZ, although enhancing the level of *Lactobacillus* genus, failed to elevate its relative abundance to above 1%, so it is unclear whether this enhancement played a strong ameliorative role. *Muribaculaceae* is the producer of SCFAs such as succinic acid, acetic acid, and propionic acid, which have important anti-inflammatory properties as well as effects on glucose and lipid homeostasis (53). Moreover, several studies have confirmed a positive correlation between the increased abundance of *Muribaculaceae* and improvement in NAFLD (54, 55). Similar results were obtained in this study that *Muribaculaceae* had wide and negative correlations with therapeutic indicators. The abundance of *Faecalibaculum* is known to be significantly elevated in NAFLD mice, which was consistent with our results (56, 57). Positive correlations were also found between *Faecalibaculum* and most therapeutic indicators. According to studies, the abundance of family *Lachnospiraceae* is significantly increased in the feces of NAFLD patients (58), some drugs reduce the abundance of *Lachnospiraceae_NK4A136_group* while improving liver injury, and this genus of bacteria is positively correlated with ALT and AST (59). This study found the positive correlations between *Lachnospiraceae_NK4A136_group* and ALT, TC and IL-6 levels. *Blautia* is a controversial genus of bacteria that has been shown to be positively correlated with serum TG levels in patients with obesity and early NAFLD (60, 61). However, as a SCFA producer, the bacteria can produce acetic acid, have a probiotic function, and can influence the colonization of specific bacteria, all of which are supported by studies (62, 63). Our results also showed that the abundance of *Blautia* was negatively correlated with ALT, TC, TG, and cytokines in NASH mice. Taken together, we speculate that *Blautia* bacteria are involved in the regulation of gut microbiota in MCD-induced NASH mice, and that the acetic acid produced by *Blautia* may delay the development of NASH, although the pros and cons of this regulation are not yet known. *Colidextribacter* reportedly showed increased abundance in hyperlipidemic animals, which is positively correlated with serum TC (64, 65). This study showed that *Colidextribacter* had positive correlations with most therapeutic indicators. So we hypothesize that its elevated abundance in NASH mice may be associated with the development of hyperlipidemia. In summary, our results demonstrated that HD-QRQZ could regulate the disordered gut microbiota in NASH mice, and this alteration may have a potential association with improved lipid metabolism. Besides, aqueous extract of *Morus alba* L. could reinforce *Muribaculaceae* while polyphenols and polysaccharides from *Morus alba* L. could increase *Lachnospiraceae_NK4A136_group* and decrease *Faecalibaculum* in mice fed with HFD (66, 67). *Coptis chinensis* Franchcan could increase the abundance of *Blautia* in ulcerative colitis rats (68). Water extract of *Anemarrhena asphodeloides* Bunge could also increase the abundance of *Blautia* in diabetic rats (69). Hydroxysafflor yellow A extracted from *Carthamus tinctorius* L. could reverse gut microbiota dysbiosis in mice fed with HFD (70). An anthraquinone-glycoside from *Rheum palmatum* L. could increase the abundance of *Lactobacillus* while decrease the abundance of *Lachnospiraceae*_*NK4A136*_*group* in T2DM rats (71). *Dioscorea oppositifolia* L. could increase the amount of *Lactobacillus* in mice with diarrhea (72). These herbs and components may exert the major regulatory effects on gut microbiota.

We then examined the effects of QRQZ on the expression of gut tight junction proteins ZO-1 and occludin in the NASH mice *via* immunohistochemistry, and also measured the serum LPS level. The results showed that QRQZ significantly increased the expression of ZO-1 and occludin in the intestine of the NASH mice, and decreased the serum LPS level. Barrier function is a fundamental role of all epithelial cells and is based on the integrity and contribution of many cytoskeletal and membrane proteins that constitute and regulate the tight-junction complexes that exist between cells (73). Studies have shown that the colon is the main site of leakage owing to disruption of the barrier function in NASH patients (74), which leads various microbial products to enter the internal environment, such as inflammation-triggering LPS. Both ZO-1 and occludin are tight junction proteins that play key roles in maintaining the integrity of the gut barrier, and animal experiments have demonstrated that elevated expression of them can reduce gut permeability (75, 76). LPS is an important component of the gut microbiota cell wall. When the gut mucosal barrier is damaged, the gut microbiota LPS can be released into the blood, which consequently aggravates the inflammatory response of the body (77).

Further study showed that QRQZ reduced important factors of the TLR4/NF-κB signaling pathway, TLR4, MyD88, p-IκB and p-NF-κBp65 in the liver of NASH mice, and downregulated the gene expression of pro-inflammatory cytokines IL-1β, IL-6, and TNF-α in the liver. Endotoxins can induce an inflammatory response by activating liver inflammatory cells. TLR4 is an important member of the Toll-like receptor family and is a receptor of LPS. LPS enters the liver and binds to TLR4, thereby promoting massive expression and activation of TLR4. Activated TLR4 then recruits MyD88 in the cells to bind to itself to form a dimer, which in turn causes NF-κB activation. NF-κB is an important nuclear transcription factor that plays an important regulatory role in the inflammatory response. NF-κB is a heterodimer composed of p50 and p65. Under normal conditions, NF-кB binds to its inhibitory protein, IκB, to form an inactive trimer that is present in the cytoplasm. MyD88 phosphorylates IκB, and the phosphorylated IκB dissociates from the NF-κB complex, which in turn leads to phosphorylation of the NF-κBp65 subunit, and the p-NF-κBp65 enters the nucleus and promotes the expression of pro-inflammatory cytokines such as IL-1β, IL-6, and TNF-α, facilitating the inflammatory response (78). A study showed that knockout of myeloid differentiation factor 2 (MD2) and TLR4 genes abrogated liver injury and inflammation in a mouse model of MCD-induced NASH (79).

**Figure 6:**
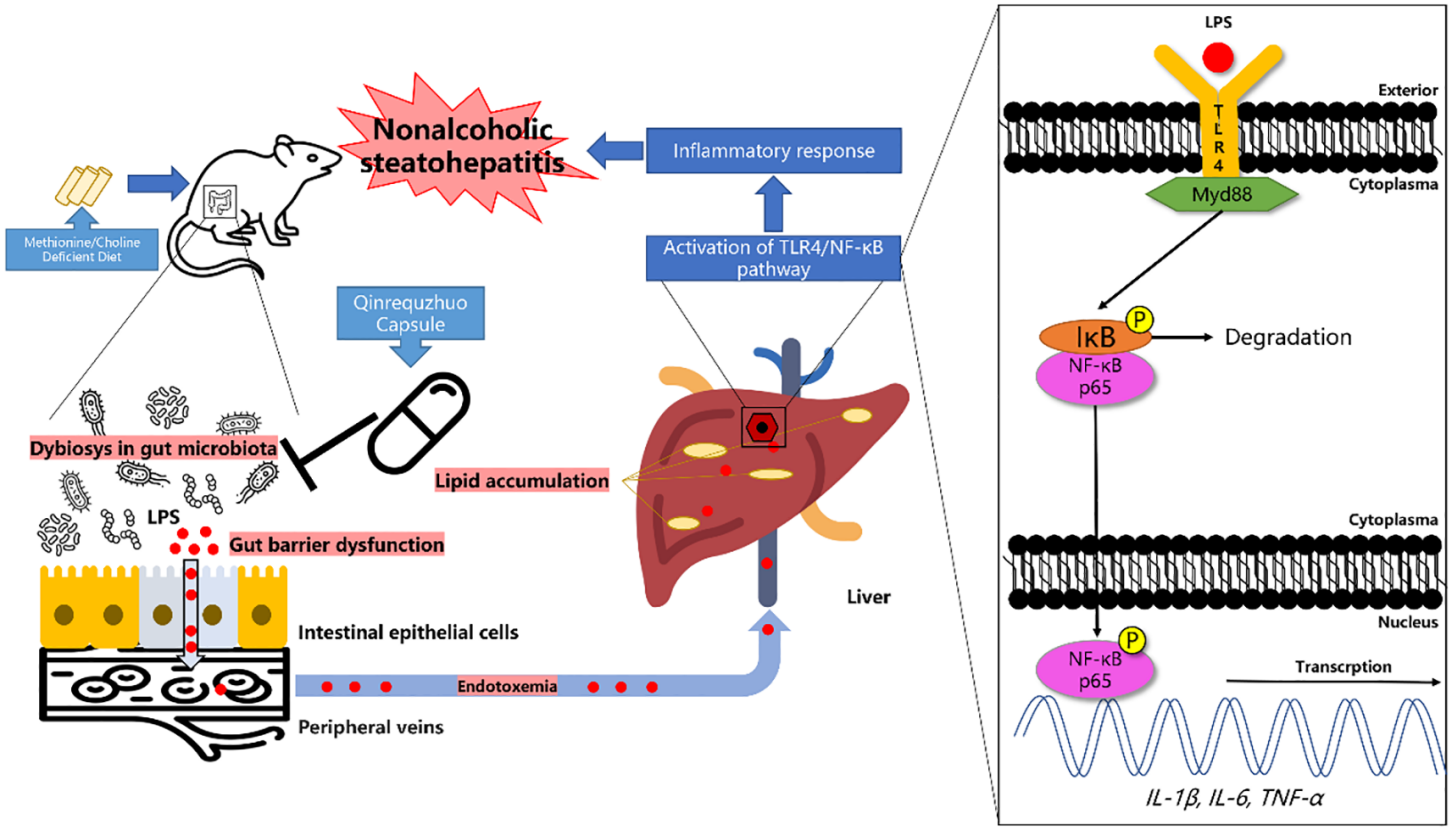
Graphical abstract.

In conclusion, our study demonstrated that QRQZ could reduce the lipid accumulation and inflammatory response in NASH model mice. The mechanisms of QRQZ on NASH were associated with modulating gut microbiota, thereby inducing the tight junction of gut barrier, reducing the endotoxemia and inhibiting the activation of TLR4/NF-κB signaling pathway in liver (**Figure 6**).

## Data availability statement

The data presented in the study are deposited in the BioProject repository, accession number PRJNA904915.

## Ethics statement

The animal study was approved by Ethics Committee of Hebei University of Chinese Medicine (Approval no. CZX2021-KY-026).

## Author contributions

SL and XS carried out the experiments and manuscript writing. SL, XW, BP and HL provided experimental help. ZZ, HZ and XS performed data analysis and result interpretation. WL supervised the experiments. YW and XS provided ideas and technical guidance for the whole work. All authors contributed to the article and approved the submitted version.
