# Supplementary material for: Qingrequzhuo capsule alleviated methionine and choline deficient diet-induced nonalcoholic steatohepatitis in mice through regulating gut microbiota, enhancing gut tight junction and inhibiting the activation of TLR4/NF-κB signaling pathway
Source: Front Endocrinol (Lausanne). 2023 Jan 19;13:1106875. doi: 10.3389/fendo.2022.1106875 (PMC9892721; doi:10.3389/fendo.2022.1106875)
Supplement: Supplementary file 1 [file Table_1.docx]

**Primer sequence**

| **Genes** | **Primer sequence (5’-3’)** |
| --- | --- |
| *TNF-α* | Forward: GATCGGTCCCCAAAGGGATG |
|  | Reverse: CCACTTGGTGGTTTGTGAGTG |
| *IL-1β* | Forward: AATGCCACCTTTTGACAGTGATG |
|  | Reverse: AGCTTCTCCACAGCCACAAT |
| *IL-6* | Forward: ACAAAGCCAGAGTCCTTCAGAG |
|  | Reverse: TCTGTGACTCCAGCTTATCTCTTG |
| *β-actin* | Forward: CCCCTGAACCCTAAGGCCA |
|  | Reverse: ATGGCTACGTACATGGCTGG |
